# Sevoflurane blocks glioma malignant development by upregulating circRELN through circRELN-mediated miR-1290/RORA axis

**DOI:** 10.1186/s12871-021-01427-1

**Published:** 2021-09-03

**Authors:** Xiaofang Kang, Hongxia Li, Zaiwang Zhang

**Affiliations:** Department of Anesthesiology, The 980 Hospital of the Joint Logistics Support Force of the Chinese People’s Liberation Army, No. 398, Zhongshan West Road, Shijiazhuang City, 050000 Hebei Province China

**Keywords:** Sevoflurane, circRELN, miR-1290, RORA, Glioma

## Abstract

**Background:**

Sevoflurane (Sev) has been reported to inhibit cancer development, and sevoflurane treatment in cancers is implicated with the deregulation of specific non-coding RNAs (ncRNAs). This study aimed to investigate the relationship between sevoflurane and circular RNA reelin (circRELN) in glioma.

**Methods:**

The expression of circRELN, microRNA-1290 (miR-1290) and RAR-related orphan receptor A (RORA) was measured by quantitative real-time PCR (qPCR). Cell proliferative capacity was assessed by cell counting kit-8 (CCK-8) and colony formation assays. Cell apoptosis and cell cycle distribution were monitored by flow cytometry assay. Cell migration was assessed by wound healing assay and transwell assay, and cell invasion was assessed by transwell assay. The protein levels of matrix metalloproteinase-2 (MMP2), MMP9 and RORA were quantified by western blot. Tumor growth in vivo was assessed by Xenograft models. The binding relationship between miR-1290 and circRELN or RORA was verified by dual-luciferase reporter assay and RNA immunoprecipitation (RIP) assay.

**Results:**

We found that circRELN expression was declined in glioma tissues and cells, while Sev treatment enhanced circRELN expression. In function, Sev notably inhibited glioma cell proliferation, migration and invasion and promoted apoptosis and cell cycle arrest, while circRELN knockdown reversed these effects. MiR-1290 served as a target of circRELN, and glioma cell malignant phenotypes recovered by circRELN knockdown were partly repressed by miR-1290 deficiency. In addition, RORA was a target of miR-1290, and glioma cell malignant phenotypes promoted by miR-1290 restoration were partly blocked by RORA overexpression. CircRELN regulated RORA expression by targeting miR-1290. In Xenograft models, Sev inhibited tumor growth by upregulating circRELN.

**Conclusion:**

Sev blocked the progression of glioma by increasing circRELN expression, and circRELN played roles in glioma partly by regulating the miR-1290/RORA network.

**Supplementary Information:**

The online version contains supplementary material available at 10.1186/s12871-021-01427-1.

## Background

Glioma is the most common primary tumor in the central nervous system of adults, accounting for about 80% of malignant primary brain tumors [1, 2]. According to its degree of malignancy, glioma is further classified into WHO-I-IV grades, and grade IV glioma is the most aggressive, invasive and lethal brain tumor in adults [3]. The challenge of high-grade glioma is the presence of highly diffuse tumor cells, which will spread to normal brain parenchyma [4]. At present, the prognosis of patients after standard of care therapies is still unsatisfactory, with a median survival time of only 12–14 months [5]. Therefore, effective therapies are urgently needed to treat this malignant tumor.

Sevoflurane (Sev) is a commonly used inhalation anesthetic. Emerging evidence indicates that Sev substantially inhibits tumor cell malignant behaviors in various cancers [6–8], hinting that Sev has potential clinical application value in the management of malignant tumors. However, little is known about the molecular mechanisms underlying Sev regulation of malignant tumors. Noticeably, Sev plays vital roles in different disorders by mediating the dysregulation of non-coding RNAs (ncRNAs), which provides new perspectives into the understanding of Sev-induced effects [9, 10]. In glioma, Sev was reported to inhibit cell cycle progression, migration and invasion by blocking the expression of circ_0012129 [11], suggesting that circular RNAs (circRNAs) might be involved in Sev-mediated regulatory networks. CircRNA is a group of ncRNAs, well-known for its closed-loop structure. CircRNAs are functional transcripts that are involved in the development of various cancers, including glioma [12, 13]. The existing microarray of circRNA expression in glioma identifies numerous circRNAs that are aberrantly expressed in tumor tissues [14]. Following the analysis of differentially expressed circRNAs in GEO dabase (GSE109569) [14], circ_0081769 (circRELN, derived from reelin (RELN) mRNA) with decreased expression in glioma tissues attracted our attention. Further analysis showed that circRELN expression was increased in Sev-treated glioma cells [14]. We hypothesized that circRELN was involved in Sev regulatory networks.

CircRNAs act as sponges of microRNAs (miRNAs) to regulate the expression of downstream mRNAs because circRNAs compete for miRNA response elements (MREs) with mRNA [15]. We therefore explored the potential mechanism of circRELN function in glioma. In this network, miR-1290 and RAR-related orphan receptor A (RORA) were screened because their functional roles had been reported in previous studies [16, 17]. However, the interactions among circRELN, miR-1290 and RORA need to be clarified to determine the action of circRELN in glioma.

Our current study investigated the expression of potential circRNAs in glioma tissues, cells and Sev-treated cells. The effects of circRELN in Sev-treated glioma cells were investigated by both gain- and loss-function assays. Moreover, the interactions among circRELN, miR-1290 and RORA were determined to reveal a new network in circRELN regulation. This study provide mechanistic insights into how Sev acts as an effective agent for glioma treatment.

## Methods

### Tissue samples

A total of 58 glioma tissues and 58 normal brain tissues (normal controls) were obtained from The 980 Hospital of the joint logistics support force of the Chinese people's Liberation Army. All samples were collected with written informed consent from patients or guardians, and the experiments had been approved by the Ethics Committee of The 980 Hospital of the joint logistics support force of the Chinese people's Liberation Army. These samples were stored at -80℃ after freezing by liquid until use.

### Cell lines and cell culture

Glioma cell lines (A172, T98G, N18 and LN229) and normal human astrocytes (NHA; control) were all purchased from Bena Culture Collection (Beijing, China). All of these cell lines were cultured at 90% DMEM (GIBCO, Grand Island, NY, USA) containing 10% fetal bovine saline (FBS; GIBCO) in a constant temperature incubator at 37℃ containing 5% CO_2_.

### Quantitative real-time PCR (qPCR)

A Trizol reagent (Invitrogen, Carlsbad, CA, USA) was used for RNA extraction. Subsequently, the one-Step SYBR PrimeScript RT-PCR Kit was used for cDNA synthesis and quantification assays for circRNAs and mRNAs according to the manufacturer’s protocols. The synthesis of cDNA from miRNAs was conducted by using a TaqMan miRNA Reverse Transcription kit (Applied Biosystems, Foster City, CA, USA), and a TaqMan Universal Master Mix II (Applied Biosystems) was applied for miRNA quantification in line with the instructions. Relative expression was obtained using the 2^−ΔΔCT^ method, using GAPDH or U6 as the internal references. All primers were listed as follows:

CircRELN: F, 5’-GACTCTTGTTATGATCTGTCCGT-3’ and R, 5’-CTGAGTAGCCAGGGTCACAT-3’; RELN: F, 5’-CGTCCTAGTAAGCACTCGCA-3’ and R, 5’-TCGCCTAAGTGACCTTCGTC-3’; miR-1290: F, 5’-GCGCGTGGATTTTTGGAT-3’ and R, 5’-AGTGCAGGGTCCGAGGTATT-3’; RORA: F, 5’-CAAAGCACAGCCCCAGTTTC-3’ and R, 5’-GCCTGTCCAGTTCGAAGACA-3’; GAPDH: F, 5’-GTCTCCTCTGACTTCAACAGCG-3’ and R, 5’-ACCACCCTGTTGCTGTAGCCAA-3’; U6: F, 5’-CTCGCTTCGGCAGCACA-3’ and R, 5’-AACGCTTCACGAATTTGCGT-3’.

### Actinomycin D and RNase R treatment

Actinomycin D treatment was carried out using actinomycin D (2 mg/mL; Sigma, St. Louis, MO, USA) to treat the experimental cells for 0 h, 4 h, 8 h, 12 h or 24 h at 37℃.

RNase R treatment was carried out using RNase R (3 U/μg; Epicentre, Madison, WI, USA) to treat the isolated RNA samples for 15 min at 37℃.

### Cell treatment

Sev (Maruishi Pharmaceutical, Osaka, Japan) was used to treat T98G and LN18 cells. In brief, cells were seeded into 6-well plates and cultured for 24 h. The plates were then placed into a sterile closed container introduced with 95% air + 5% CO_2_ containing different concentrations of Sev (1.7, 3.4, and 5.1%) [18]. Sev was delivered into a container using an anesthesia vaporizer. The changes of Sev concentrations were monitored using a gas analyzer (Ohmeda 5250 RGM, Louisville, CO, USA).

### Cell transfection

Small interference RNA (siRNA) for circRELN knockdown (si-circRELN) and its negative control (si-NC) were provided by Genomeditech (Shanghai, China). The mimics for miR-1290 upregulation (miR-1290), the inhibitors for miR-1290 inhibition (anti-miR-1290) and their negative controls (miR-NC and anti-miR-NC) were all obtained from Ribobio (Guangzhou, China). PCDNA3.1 for RORA overexpression (pcDNA-RORA) and its control blank vector (pcDNA) were constructed by Genepharma (Shanghai, China). Oligonucleotides or vectors were transfected into cells using Lipofectamine 3000 reagent (Invitrogen).

### Cell counting kit-8 (CCK-8) assay

After transfection, cells were plated into 96-well plates (2,000 cells/well) and maintained for 24 h. Then, 10 µL CCK-8 reagent (Dojindo, Kumamoto, Japan) was pipetted into each well for incubation for 2 h. The value of 450 nm optical density (OD) was detected using SpectraMax M5 (Molecular Devices, San Jose, CA, USA).

### Colony formation assay

After transfection, cells were plated into 6-well plates (300 cells/well) and cultured for 2 weeks. Colonies (over 200 cells) were washed with phosphate buffered saline (PBS; Sigma), fixed with methanol and stained with crystal violet (Sigma). The number of colonies was counted under a microscope (Nikon, Tokyo, Japan).

### Flow cytometry assay

Cells with treatment or transfection were cultured for 48 h and collected by Trypsin digestion. Cells were then washed with PBS and used for apoptosis detection using the Annexin V-FITC/propidium iodide (PI) apoptosis detection kit (Sigma). In brief, cells were suspended in binding buffer and then treated with 5 µL Annexin V-FITC and 10 µL PI. Cells were incubated in the dark for 15 min, and cell apoptosis was analyzed using a FACScan flow cytometry (BD Biosciences, Franklin Lakes, NJ, USA).

Cells with treatment or transfection were cultured for 24 h and collected by Trypsin digestion. Cells were washed with PBS and fixed in 70% ethanol at 4℃ overnight. Next, cells were washed with PBS and stained with PI/RNase A working buffer (BD Biosciences) in the dark for 15 min. Cell cycle distribution at various phases was determined using a FACScan flow cytometry (BD Biosciences).

### Wound healing assay

After transfection, cells were plated in 6-well plates until 90% confluence. An artificial wound was created using a 200-μL pipette tip. The photos of wound closure were recorded at 0 and 24 h. Wound healing rate was measured using Image J software (NIH, Bethesda, MA, USA).

### Transwell assay

Matrigel-coated transwell chambers (BD Biosciences) were used for cell invasion analysis, while non-treated chambers were used for cell migration analysis. Cells after transfection were suspended by FBS-free DMEM and transferred in the top of chambers, and DMEM containing 10% FBS was added to the bottom of chambers as a chemoattractant. After 24-h incubation, cells still in the upper chambers were removed by cotton swabs, and cells in the lower surface were fixed with methanol and stained with crystal violet (Sigma). The images of migration or invasion were recorded using a microscope (100 × ; Nikon).

### Western blot

Total proteins extracted using RIPA buffer (Roche, Mannheim, Germany) were quantified by BCA kit (Roche). Then, 30 µg proteins were separated and membrane-transferred by standard western blot procedures. The primary antibodies, including matrix metalloproteinase-2 antibody (anti-MMP2; ab86607; Abcam, Cambridge, MA, USA), matrix metalloproteinase-9 antibody (anti-MMP9; ab137867; Abcam), anti-RORA (ab70061; Abcam) and anti-GAPDH (ab181602; Abcam), and the secondary antibodies (ab205718 and ab505719; Abcam) were used to probe proteins. The Western Chemiluminescent HRP Substrate (Millipore, Billerica, MA, USA) was utilized to visualize the protein signals. The expression data were quantified by using Image J software (version 1.46; NIH, Bethesda, MA, USA).

### Dual-luciferase reporter assay

The binding relationship between circRELN and miR-1290 was analyzed by circinteractome (https://circinteractome.nia.nih.gov/) and circbank (http://www.circbank.cn/). The binding relationship between miR-1290 and RORA was analyzed by Targetscan (http://www.targetscan.org/vert_72/).

First, the wild-type (WT) and mutant-type (MUT) reporter plasmids of circRELN and RORA 3’UTR were constructed, naming as circRELN WT, circRELN MUT, RORA 3’UTR WT and RORA 3’UTR MUT. T98G and LN18 cells were cotransfected with miR-1290 or miR-NC and one of these plasmids. Cells were then incubated for 48 h, and luciferase activity in cells was detected using the dual-luciferase reporter assay kit (Promega, Madison, WI, USA).

### RNA immunoprecipitation (RIP) assay

RIP assay was performed to determine the binding relationship using the Magna RIP RNA-Binding Protein Immunoprecipitation Kit (Millipore) according to the manufacturer’s instructions. The magnetic beads were conjugated with antibodies against Ago2 (Millipore) or IgG (control; Millipore). Purified RNAs from beads were extracted and analyzed by qPCR.

### Xenograft models

The animal experiment was conducted in accordance with the Animal Care and Use Committee of The 980 Hospital of the joint logistics support force of the Chinese people's Liberation Army. Short hairpin RNA (shRNA) for circRELN knockdown (sh-circRELN) and its negative control (sh-NC) were packaged by lentivirus by Genepharma. Nude mice (BALB/c, 6-week-old, female, *n* = 20) were purchased from Beijing Vital River Laboratory Animal Technology (Beijing, China) and randomly divided into 4 groups (*n* = 5 per group), including control (T98G cell injection), Sev (T98G cell injection and Sev administration), Sev + sh-NC (T98G cell infected with sh-NC injection and Sev administration) and Sev + sh-circRELN (T98G cell infected with sh-circRELN injection and Sev administration). T98G cells were injected into nude mice by subcutaneous injection. For Sev administration, T98G cells were treated with 5.1% Sev as mentioned above and then injected into mice. Tumor volume (length × width^2^ × 0.5) was measured once a week. Tumor growth was allowed to develop for 35 days. After that, all mice were administrated with anesthetics and then sacrificed by cervical dislocation. Tumor tissues were excised for other analyses.

### Statistical analysis

Data were expressed as mean ± standard deviation (SD). Student’s *t*-test or analysis of variance (ANOVA) was performed to compare differences between two groups or among multiple groups. GraphPad Prism 7.0 (GraphPad, La Jolla, CA, USA) was used for statistical analysis and graph making. *P* value < 0.05 was considered statistically significant.

## Results

### CircRELN was poorly expressed in glioma tumor tissues and cell lines

**Fig. 1:**
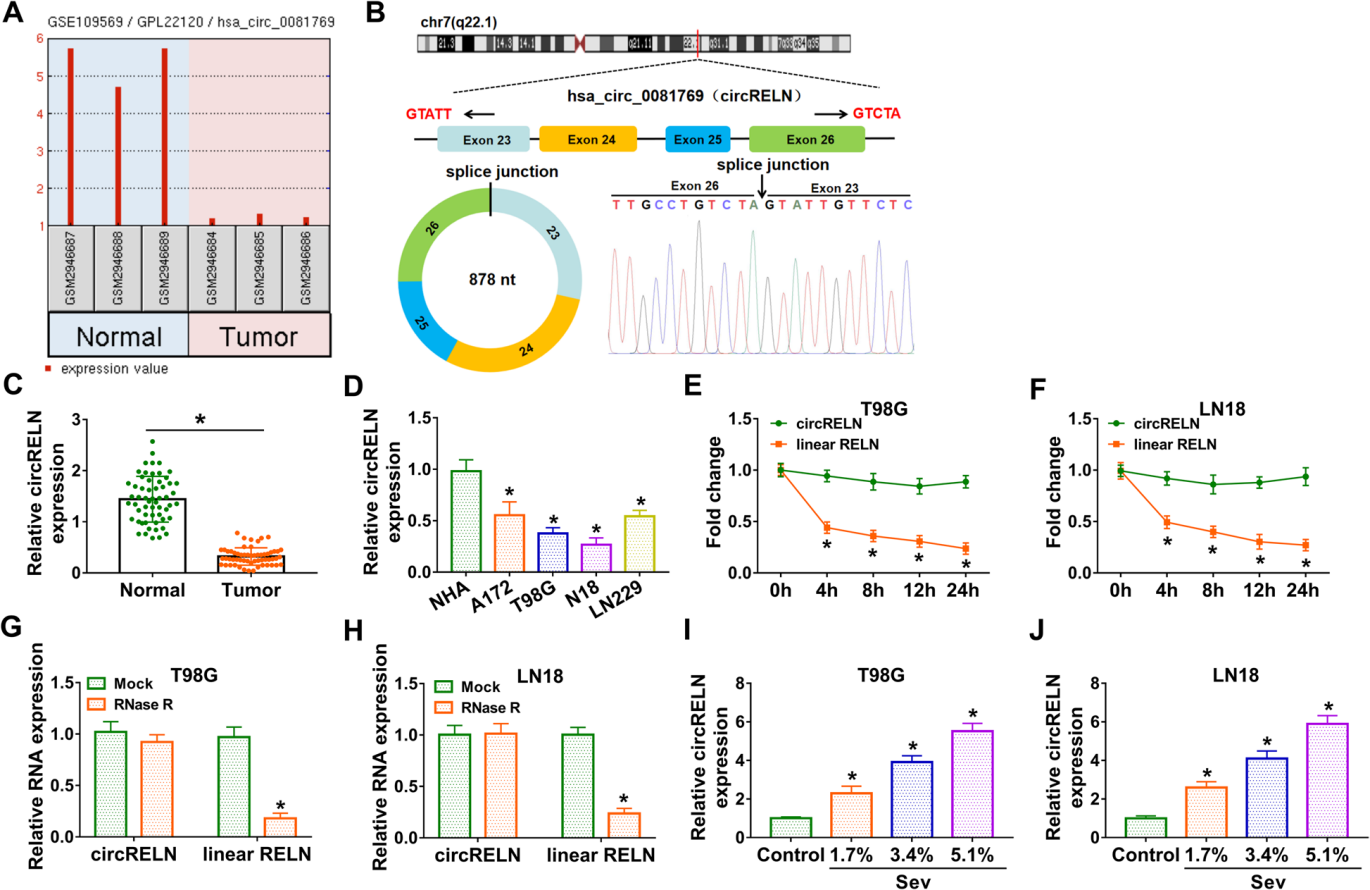
CircRELN was downregulated in glioma tissues and cells. **A** The data of circ_0081769 expression was obtained from GSE109569 dataset. **B** Schematic diagram illustrated the formation of circ_0081769 (circRELN). **C** The expression of circRELN in tissue samples was detected by qPCR. **D** The expression of circRELN in cell lines was detected by qPCR. **E and F** The circularity and stability of circRELN were examined by actinomycin D. **G and H** The circularity and stability of circRELN were examined by RNase R. **I and J** The expression of circRELN in Sev-treated T98G and LN18 cells was detected by qPCR. *P < 0.05

The data from GSE109569 dataset displayed that hsa_circ_0081769 (circRELN) expression was notably decreased in tumor tissues of glioma compared to normal tissues (Fig. 1A). CircRELN was derived from the exon 23-exon 26 regions of RELN mRNA, with 878 nucleotides in length, which was verified by Sanger sequencing (Fig. 1B). In our samples, we found that relative expression of circRELN in glioma tumor tissues (*N* = 58) was significantly declined compared with that in normal controls (*N* = 58) (Fig. 1C). Likewise, the expression of circRELN was also decreased in glioma cell lines (A172, T98G, N18 and LN229) compared with that in NHA cells (Fig. 1D). T98G and N18 cells were used in the follow-up assay because a lower expression level of circRELN was shown in these two cell lines. In T98G and N18 cells treated with actinomycin D, the expression of linear RELN was strikingly decreased, while the expression of circRELN was hardly changed (Fig. 1E and F). Besides, compared to linear RELN, circRELN was resistant to RNase R digestion (Fig. 1G and H). In T98G and LN18 cells treated with Sev, we found that the expression of circRELN was strikingly increased in a dose-dependent manner (Fig. 1I and J). These data suggested that circRELN was downregulated in glioma tissues and cells, and Sev promoted the expression of circRELN.

### Sev administration inhibited proliferation, migration and invasion and induced apoptosis and cell cycle arrest in T98G and LN18 cells by increasing circRELN expression

**Fig. 2:**
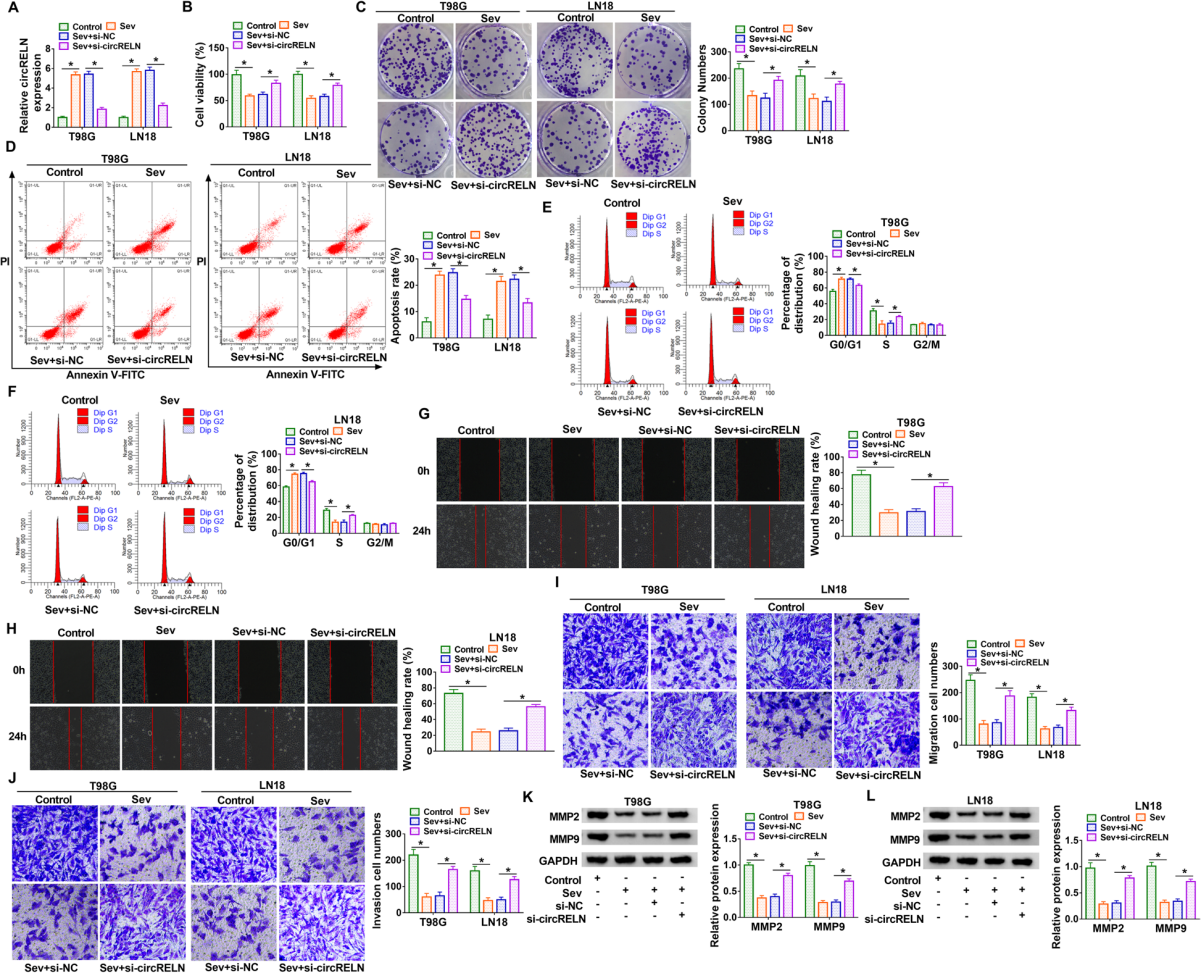
Sev inhibited malignant cell behaviors in T98G and LN18 cells by upregulating circRELN. Experimental cells in this section included control, Sev, Sev + si-circRELN or Sev + si-NC treatment. **A** The expression in experimental cells was detected by qPCR. **B** Cell viability was checked by CCK-8 assay. **C** Cell formation ability was checked by colony formation assay. **D** Cell apoptosis was monitored using flow cytometry assay. **E and F** Cell cycle progression was monitored using flow cytometry assay. (G and H) Cell migration was assessed by wound healing assay. **I and J** Cell migration and cell invasion were assessed by transwell assay. **K and L** The expression of MMP2 and MMP9 was measured by western blot. *P < 0.05

Given that circRELN was induced by Sev, we thus reduced circRELN expression in Sev-treated T98G and LN18 cells using siRNA knockdown to explore the function of circRELN. The expression was notably strengthened in Sev-treated T98G and LN18 cells, while si-circRELN transfection largely reduced circRELN expression (Fig. 2A). In function, CCK-8 assay presented that Sev treatment significantly impaired cell viability, while si-circRELN transfection partly restored cell viability (Fig. 2B). The ability of colony formation suppressed by Sev was partly recovered by circRELN knockdown (Fig. 2C). Besides, flow cytometry assay displayed that Sev-induced cell apoptosis was largely inhibited by circRELN knockdown (Fig. 2D). Sev treatment induced cell cycle arrest at the G0/G1 transition to S phase, while circRELN knockdown alleviated this arrest (Fig. 2E and F). Wound healing assay exhibited that the ability of cell migration was blocked by Sev treatment but largely recovered by circRELN knockdown (Fig. 2G and H). Transwell assays showed that Sev treatment significantly reduced the number of migrated or invaded cells, while si-circRELN transfection pronouncedly increased the number of migrated or invaded cells (Fig. 2I and J). MMP2 and MMP9 were markers closely associated with tumor invasion and metastases. Herein, we found that the protein levels of MMP2 and MMP9 were depleted by Sev but partly restored by circRELN knockdown (Fig. 2K and L). Overall, a series of cellular malignant behaviors in T98G and LN18 cells were alleviated by Sev but recovered by circRELN knockdown.

### MiR-1290 was verified to be a target of circRELN

**Fig. 3:**
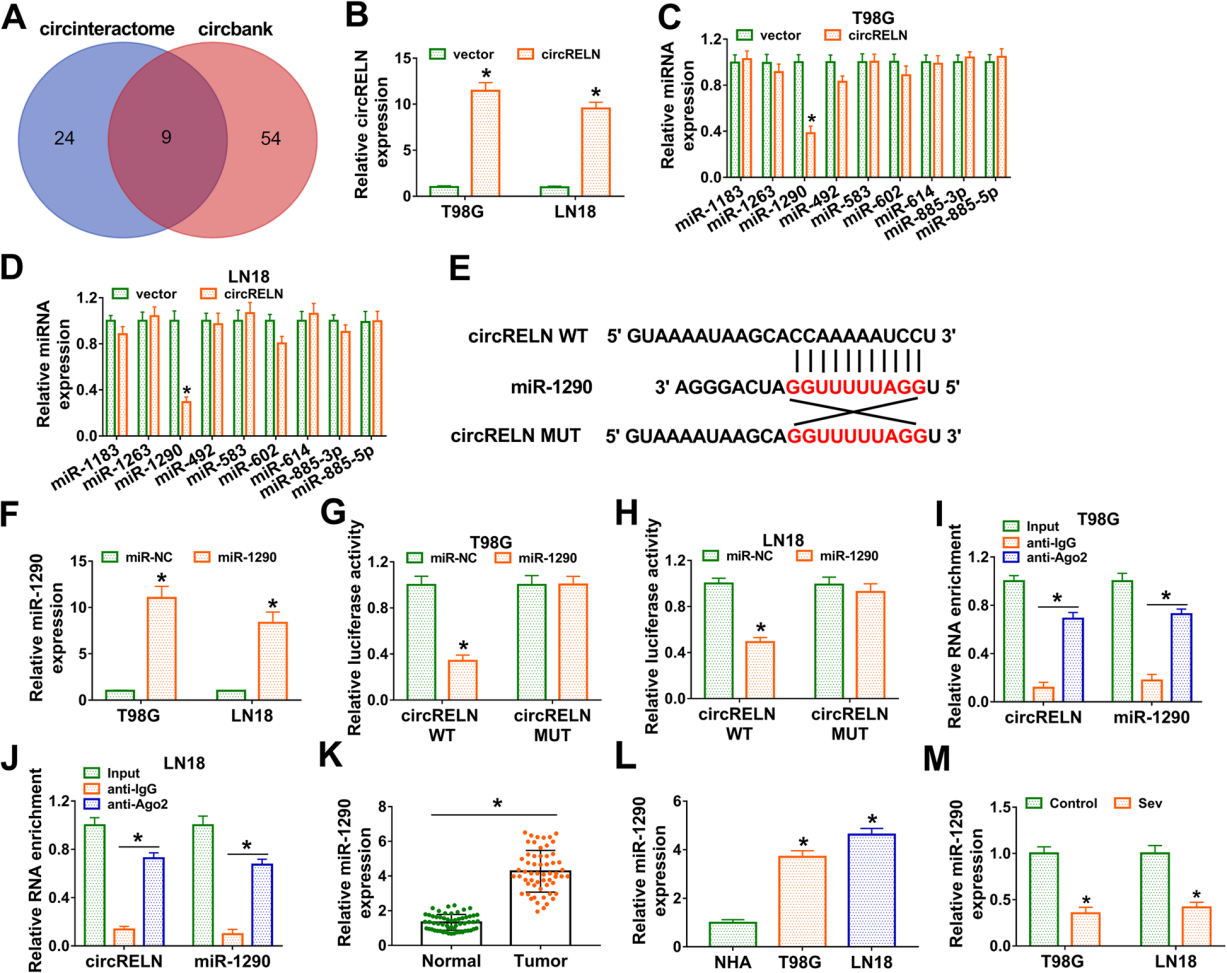
MiR-1290 was a target of circRELN. **A** The potential target miRNAs were predicted by circinteractome and circbank. **B** The efficiency of circRELN overexpression was checked by qPCR. **C and D** The expression of predicted target miRNAs in T98G and LN18 cells was checked by qPCR. **E** The binding site between circRELN and miR-1290 was shown. **F** The efficiency of miR-1290 mimic was checked by qPCR. **G and H** The binding relationship between circRELN and miR-1290 was verified by dual-luciferase reporter assay. **I and J** The binding relationship between circRELN and miR-1290 was verified by RIP assay. **K** The expression of miR-1290 in tissue samples was detected by qPCR. **L** The expression of miR-1290 in cell lines was detected by qPCR. (M) The expression of miR-1290 in Sev-treated T98G and LN18 cells was detected by qPCR. *P < 0.05

To explore the functional mechanism of circRELN, we analyzed the target miRNAs of circRELN. The data from circinteractome and circbank databases showed that there were a total of 9 miRNAs collectively detected in both two databases (Fig. 3A). We transfected circRELN into T98G and LN18 cells and found that the expression of circRELN was strikingly increased (Fig. 3B). In circRELN-overexpressed T98G and LN18 cells, the expression of miR-1290 was remarkably decreased, while other miRNAs expression had no noticeable difference (Fig. 3C and D). The binding site between circRELN and miR-1290 and the mutated binding site were shown in Fig. 3E. We found that miR-1290 transfection efficiently promoted the expression of miR-1290 (Fig. 3F). Reduced firefly luciferase expression indicates the binding of endogenous or introduced miRNAs to the cloned miRNA target sequence. The data from dual-luciferase reporter assay presented that miR-1290 significantly reduced luciferase activity in T98G and LN18 cells transfected with circRELN WT rather than circRELN MUT (Fig. 3G and H). Subsequent RIP assay presented that circRELN and miR-1290 could interact with Ago2 binding protein compared to IgG (Fig. 3I and J), which further verified the binding relationship between circRELN and miR-1290. MiR-1290 expression was strikingly elevated in glioma tissues and cell lines (T98G and LN18) relative to normal tissues and NHA cells, respectively (Fig. 3K and L). In addition, Sev treatment significantly impaired the expression of miR-1290 (Fig. 3M). Overall, all data demonstrated that miR-1290 was a target of circRELN.

### CircRELN knockdown recovered cellular malignant behaviors by enriching miR-1290 in Sev-treated T98G and LN18 cells

**Fig. 4:**
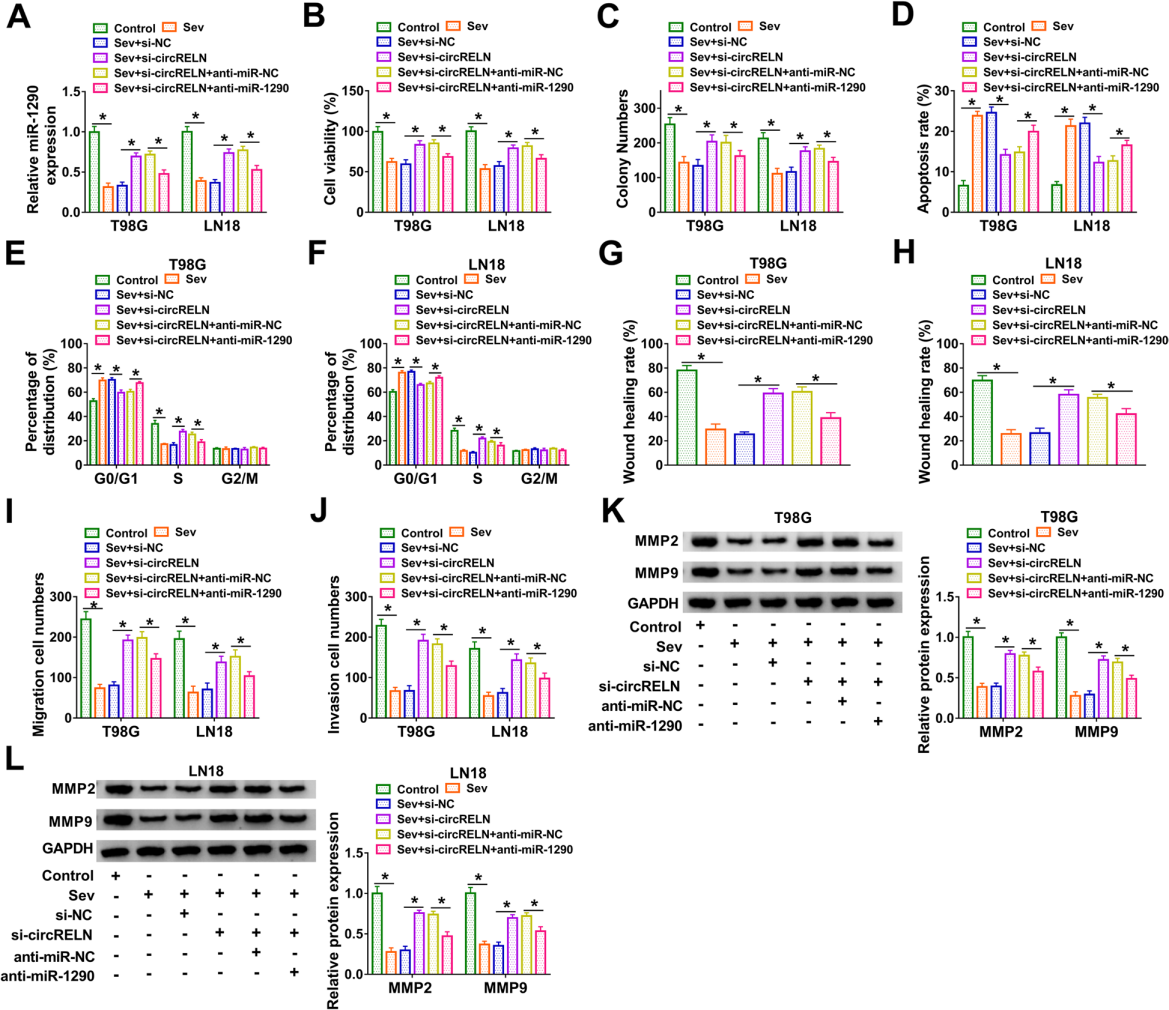
CircRELN knockdown recovered cellular malignant behaviors by enriching miR-1290 in Sev-treated T98G and LN18 cells. Experimental cells in this section included Control, Sev, Sev + si-NC, Sev + si-circRELN, Sev + si-circRELN + anti-miR-NC or Sev + si-circRELN + anti-miR-1290 treatment. **A** The expression of miR-1290 was measured by qPCR. **B and C** Cell proliferation was assessed by CCK-8 assay and colony formation assay. **D** Cell apoptosis was assessed by flow cytometry assay. **E and F** Cell cycle progression was analyzed by flow cytometry assay. **G and H** Cell migration was assessed by wound healing assay. (**I and J** Cell migration and cell invasion were assessed by transwell assay. **K and L** The protein levels of MMP2 and MMP9 were detected by western blot. *P < 0.05

We explored the interactions between circRELN and miR-1290 in T98G and LN18 cells. The expression of miR-1290 depleted in Sev-treated cells was partly recovered by alone si-circRELN transfection but repressed by si-circRELN + anti-miR-1290 cotransfection (Fig. 4A). In terms of function, CCK-8 assay showed that cell viability in Sev-treated cells was recovered by si-circRELN transfection, and miR-1290 inhibition partly inhibited cell viability (Fig. 4B). The ability of colony formation was recovered in Sev-treated cells transfection with si-circRELN but repressed in cells transfected with si-circRELN + anti-miR-1290 (Fig. 4C). In contrast, the cotransfection of si-circRELN + anti-miR-1290 largely recovered cell apoptosis rate and cell cycle arrest that were alleviated by alone si-circRELN transfection in T98G and LN18 cells (Fig. 4D-F). Wound healing assay showed that cell migration was promoted in Sev-treated cells transfected with si-circRELN but partly repressed in Sev-treated cells transfected with si-circRELN + anti-miR-1290 (Fig. 4G and H). Transwell assay showed that cell migration and cell invasion were promoted in Sev-treated cells transfected with si-circRELN but partly blocked in Sev-treated cells transfected with si-circRELN + anti-miR-1290 (Fig. 4I and J). Sev suppressed the expression of MMP2 and MMP9 that was partially restored by the knockdown of circRELN. The effect of the circRELN knockdown on MMP2 and MMP9 expression was further attenuated by MiR-1290 inhibition (Fig. 4K and L). The data showed that circRELN knockdown recovered cellular malignant behaviors by enriching miR-1290.

### CircRELN regulated RORA expression by depleting miR-1290

**Fig. 5:**
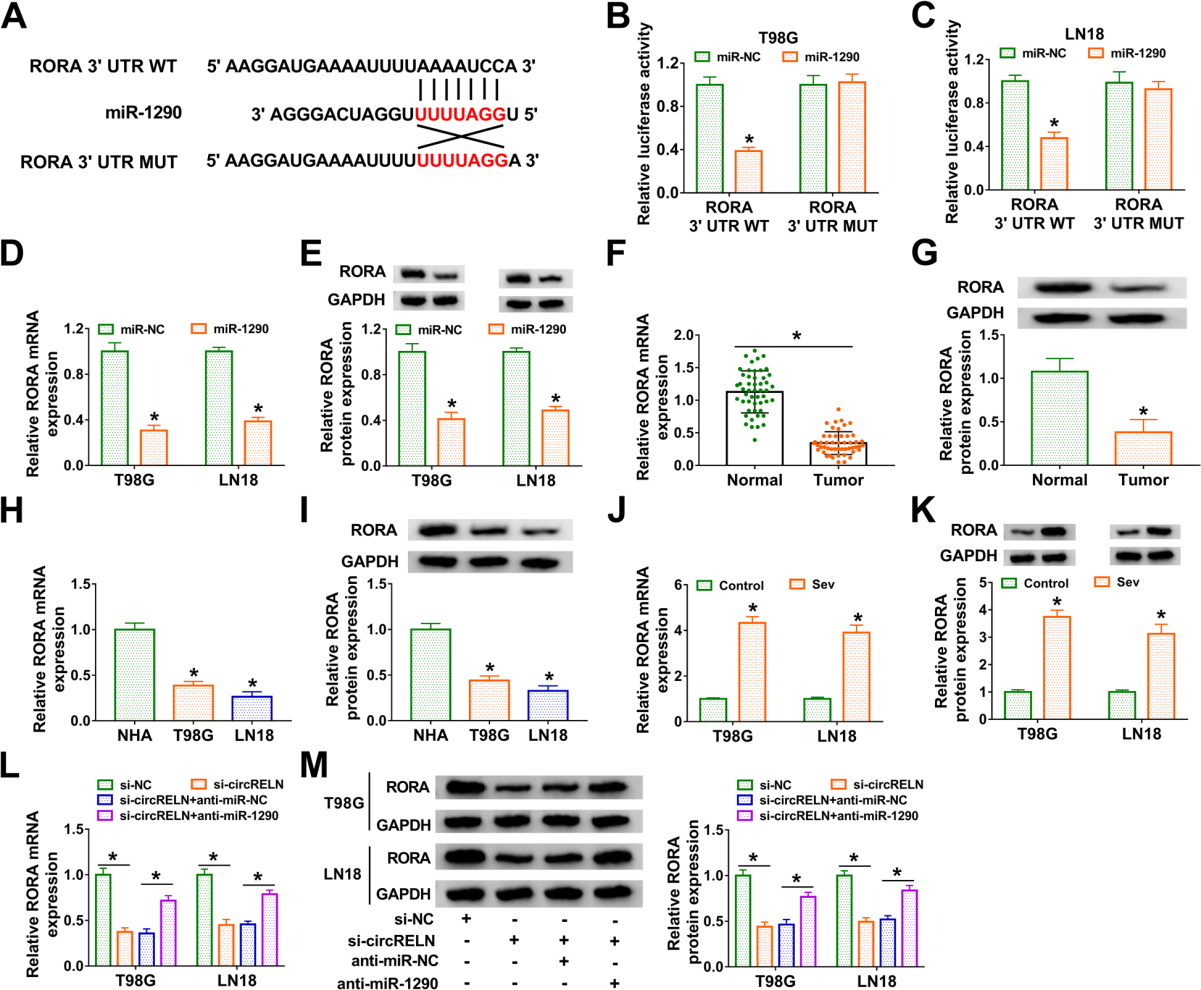
CircRELN regulated RORA expression by targeting miR-1290. **A** The binding site between RORA 3’UTR and miR-1290 was analyzed by Targetscan. **B and C** The binding relationship between RORA and miR-1290 was confirmed by dual-luciferase reporter assay. **D and E** The expression of RORA in T98G and LN18 cells with miR-1290 enrichment was measured by qPCR and western blot. **F and G** The expression of RORA in tissue samples was measured by qPCR and western blot. **H and I** The expression of RORA in cell lines was measured by qPCR and western blot. **J and K** The expression of RORA in Sev-treated T98G and LN18 cells was detected by qPCR and western blot. **L and M** The expression of RORA in T98G and LN18 cells transfected with si-circRELN, si-NC, si-circRELN + anti-miR-NC or si-circRELN + anti-miR-NC was detected by qPCR and western blot. *P < 0.05

We next analyzed the target mRNAs of miR-1290. The data from Targetscan database showed that miR-1290 directly interacted with RORA 3’UTR, the binding site was shown in Fig. 5A. Dual-luciferase reporter assay displayed that miR-1290 markedly reduced luciferase activity in T98G and LN18 cells transfected with RORA 3’UTR WT rather than RORA 3’UTR MUT (Fig. 5B and C). In addition, the expression of RORA was remarkably decreased in T98G and LN18 cells transfected with miR-1290 at both mRNA and protein levels (Fig. 5D and E). The expression of RORA was notably downregulated in glioma tumor tissues compared to normal tissues (Fig. 5F and G). Also, the expression of RORA was decreased in T98G and LN18 cells compared with that in NHA cells (Fig. 5H and I). Moreover, the expression of RORA was significantly elevated in Sev-treated T98G and LN18 cells (Fig. 5J and K). More importantly, we found that RORA expression was notably decreased in T98G and LN18 cells with circRELN knockdown, while additional miR-1290 inhibition enhanced RORA expression (Fig. 5L and M). These data demonstrated that RORA was a target of miR-1290, and circRELN regulated RORA expression by targeting miR-1290.

### MiR-1290 aggravated Sev-inhibited cellular malignant behaviors by depleting RORA

**Fig. 6:**
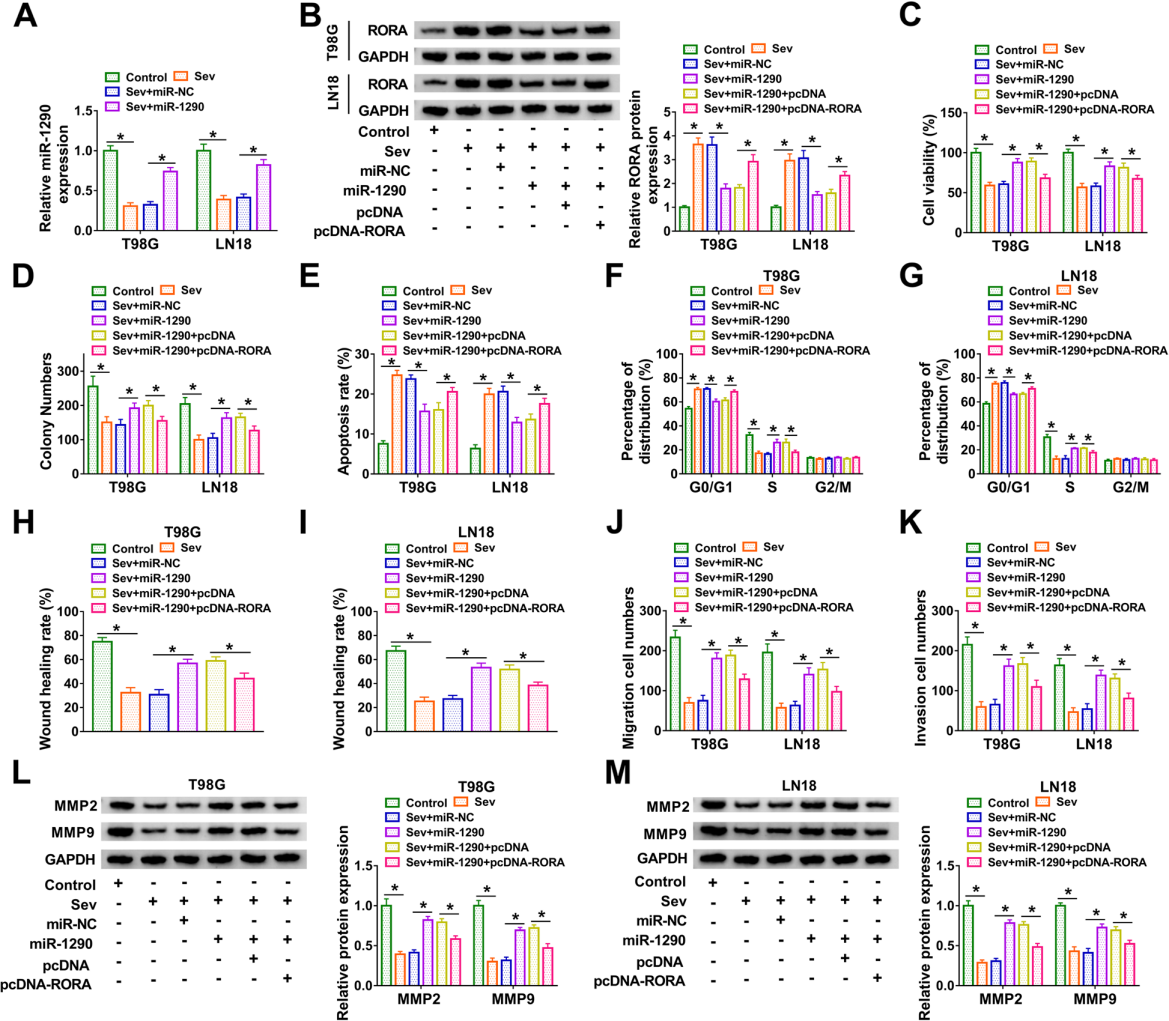
MiR-1290 aggravated Sev-inhibited cellular malignant behaviors by depleting RORA. Experimental cells in this section included control, Sev, Sev + miR-1290, Sev + miR-NC, Sev + miR-1290 + pcDNA-RORA or Sev + miR-1290 + pcDNA. **A** The expression of miR-1290 in Sev-treated T98G and LN18 cells transfected with miR-1290 or miR-NC was detected by qPCR. **B** The expression of RORA in these experimental cells was detected by western blot. **C and D** Cell proliferation was assessed by CCK-8 assay and colony formation assay. **E** Cell apoptosis was monitored by flow cytometry assay. **F and G** Cell cycle progression monitored by flow cytometry assay. **H and I** Cell migration was assessed by wound healing assay. **J and K** Cell migration and cell invasion were assessed by transwell assay. **L and M** The protein levels of MMP2 and MMP9 were measured by western blot. *P < 0.05

The expression of miR-1290 was depleted in Sev-treated T98G and LN18 cells but recovered in Sev-treated cells transfected with miR-1290 (Fig. 6A). The expression of RORA was significantly reduced in Sev-treated cells transfected with miR-1290 but restored in Sev-treated cells transfected with miR-1290 + pcDNA-RORA (Fig. 6B). In terms of function, Sev-depleted cell viability and colony formation ability were recovered by miR-1290 restoration, while RORA reintroduction repressed cell viability and colony formation ability (Fig. 6C and D). Besides, Sev-induced cell apoptosis and cell cycle arrest were blocked by miR-1290 restoration but restored by RORA reintroduction (Fig. 6E-G). In addition, cell migration detected by wound healing assay and transwell assay was promoted by miR-1290 in Sev-treated T98G and LN18 cells but repressed by miR-1290 + pcDNA-RORA (Fig. 6H-J). Cell invasion was also promoted by miR-1290 but impaired by miR-1290 + pcDNA-RORA in Sev-treated T98G and LN18 cells (Fig. 6K). Additionally, the protein levels of MMP2 and MMP9 inhibited in T98G and LN18 cells were recovered by miR-1290 but repressed by miR-1290 + pcDNA-RORA (Fig. 6L and M). Overall, miR-1290 enhanced Sev-inhibited cellular malignant behaviors by depleting RORA.

### CircRELN downregulation inhibited tumor growth in vivo

**Fig. 7:**
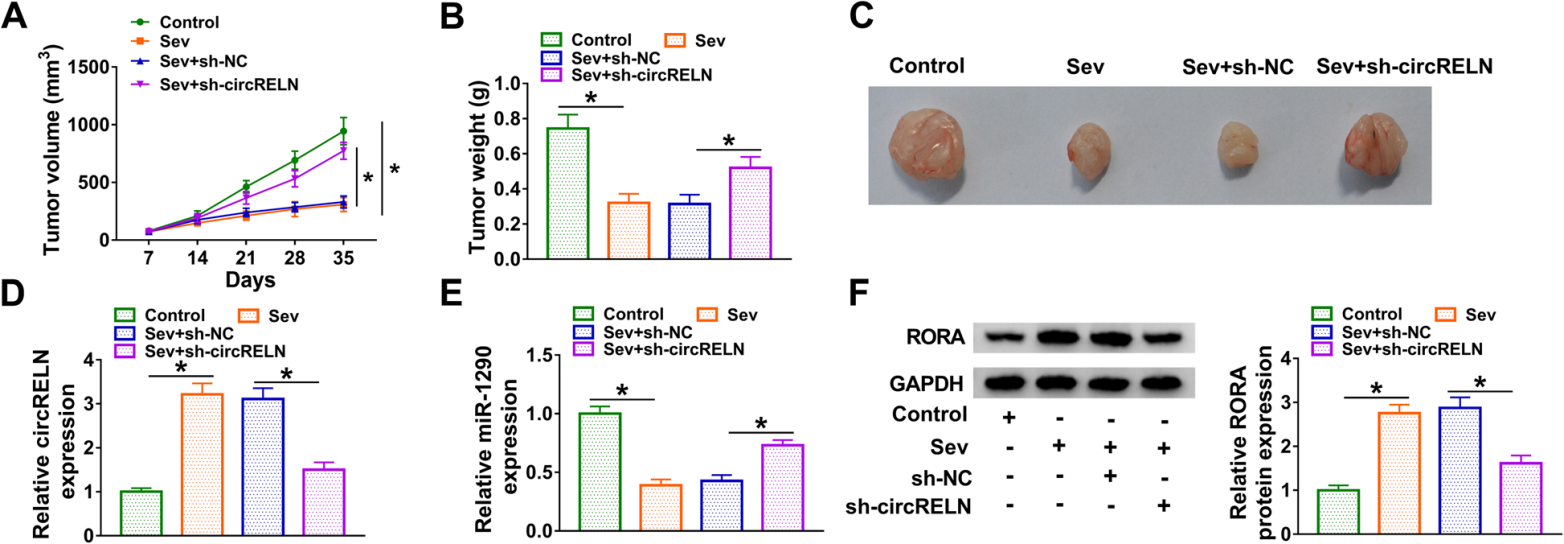
CircRELN downregulation inhibited tumor growth in vivo. **A and B** Tumor volume and tumor weight were measured to observe tumor growth. **C** Tumor tissues were excised, and representative images of tumor size were shown. **D and E** The expression of circRELN and miR-1290 in the excised tissues was detected by qPCR. **F** The expression of RORA in the excised tissues was detected by western blot. *P < 0.05

T98G cells infected with lentivirus-mediated sh-circRELN or sh-NC were injected into nude mice to observe the effects on tumor growth in vivo. As results, we found Sev treatment significantly inhibited tumor growth compared to control, while sh-circRELN promoted tumor growth compared to sh-NC, which was concluded from tumor volume, tumor weight and tumor size (Fig. 7A-C). Further analysis showed that circRELN expression was increased in Sev-administered group, while circRELN expression was declined in Sev + sh-circRELN-administered group (Fig. 7D). MiR-1290 expression was significantly declined in Sev-treated group, while its expression was enhanced in Sev + sh-circRELN-administered groups (Fig. 7E). The expression of RORA in different group was consistent with circRELN expression (Fig. 7F). The data suggested that circRELN downregulation governed the miR-1290/RORA axis to inhibit tumor growth in vivo.

## Discussion

In the present study, we mainly discovered that circRELN was significantly downregulated in glioma tissues and cells. However, Sev treatment notably increased the expression of circRELN in glioma cells. Functional assays presented that Sev inhibited glioma cell proliferation, migration and invasion and promoted apoptosis and cell cycle arrest, while circRELN knockdown partly abolished these effects. Further analysis showed that circRELN played biological functions in glioma by upregulating RORA via competitively targeting miR-1290, which was demonstrated by rescue experiments. Additionally, Sev administration was shown to suppress tumor growth in vivo, while circRELN downregulation reversed the role of Sev. These findings illustrated the functional mechanism of Sev in glioma in a new insight.

Sev is a new type of halogen inhaled anesthetic. A growing number of studies have demonstrated the efficacy and safety of Sev [19], and Sev has been shown to have cardioprotective effects and protective effects in other organs [20]. Emerging topics focus on the potential capacity of Sev in tumor inhibition. For example, Sev blocked the growth and migration of cervical cancer, colon cancer and ovarian cancer via targeting numerous oncogenic pathways [8, 21, 22]. Sev was documented to inhibit glioma cell proliferation and invasion and induce apoptosis by degrading multiple oncogenes [23, 24]. Besides, recent studies introduced that Sev blocked the progression of glioma by targeting the circRNA regulatory networks [11, 25], suggesting that Sev had pivotal roles in mediating the deregulation of circRNAs. In our study, we found that circRELN expression, decreased in glioma tissues and cells, was inversely enhanced in Sev-treated glioma cells. Functionally, Sev-suppressed glioma cell malignant behaviors were recovered by circRELN knockdown, suggesting that Sev impeded the development of glioma by upregulating circRELN. Low expression of circRELN was previously shown in glioma tissues in circRNA expression profile [14]. The detailed functions of circRELN were still unclear. Herein, we, for the first time, investigated the role of circRELN in Sev-administered glioma in vitro and in vivo, and we concluded that circRELN knockdown recovered numerous cell malignant behaviors, including proliferation, cycle progression, migration and invasion.

Subsequently, we explored the functional networks of circRELN in glioma. By the analysis of bioinformatics tools, numerous putative targets of circRELN were obtained, and only miR-1290 was significantly downregulated by circRELN overexpression. In addition, the binding between circRELN and miR-1290 was further verified by dual-luciferase reporter assay and RIP assay. We displayed that miR-1290 expression was upregulated in glioma tissues and cells, which was consistent with a previous study [16]. MiR-1290 was reported to promote the capacities of proliferation, migration and invasion of glioma cells [16]. Besides, the oncogenic effects of miR-1290 were mentioned in multiple cancers, such as gastric cancer, non-small cell lung cancer and colorectal cancer [26–28]. Consistent with these ideas, we discovered that miR-1290 inhibition repressed glioma cell malignant behaviors that were recovered by circRELN knockdown in Sev-treated cells, while miR-1290 enrichment restored these cell malignant behaviors that were inhibited Sev, suggesting that miR-1290 might attenuate the effects of Sev.

Moreover, the targets of miR-1290 were identified. Among these targets, we found that RORA was widely investigated in previous studies and associated with cancer progression [17, 29]. These studies demonstrated that RORA was a tumor suppressor to block cancer malignant progression. Then, the binding relationship between miR-1290 and RORA was confirmed by dual-luciferase reporter assay, and rescue experiments showed that RORA overexpression reversed the effects of miR-1290 restoration, suggesting that RORA was a downstream target of miR-1290. The role of RORA was also partly explored in glioma, and RORA overexpression inhibited proliferative capacity and tumorigenesis in glioma [17]. Our study exhibited consistent results and showed that RORA overexpression repressed cell proliferation, migration, invasion and induced apoptosis and cell cycle arrest in glioma.

Our present study provided a new mechanism for Sev to inhibit glioma development and further enriched the role of circRELN in mediating the effect of agents in glioma progression. These could greatly deepen the understanding of glioma pathogenesis. Even so, there are still limitations in our current study, for example, other underlying miRNA-mRNA networks have not been identified. Besides, it is not clear whether there are oncogenic pathways involved in circRELN-mediated miR-1290/RORA regulatory network. Future work should focus on other regulatory networks associated with circRELN to further clarify the role of circRELN in Sev-inhibited glioma progression.

## Conclusions

In summary, Sev plays an important role in blocking the development of glioma by increasing the expression of circRELN, and we find that circRELN governs the miR-1290/RORA axis in its networks. Overall, we define that Sev blocks glioma malignant development by upregulating circRELN through circRELN-mediated miR-1290/RORA axis.

## Supplementary Information

**Additional file 1.**

## Data Availability

All data generated or analysed during this study are included in this published article [and its supplementary information files].

## Abbreviations

Sev: Sevoflurane
ncRNAs: Non-coding RNAs
circRELN: Circular RNA reelin
miR-1290: MicroRNA-1290
CCK-8: Cell counting kit-8
MMP2: Matrix metalloproteinase-2
