# Supplementary material for: Sevoflurane blocks glioma malignant development by upregulating circRELN through circRELN-mediated miR-1290/RORA axis
Source: BMC Anesthesiol. 2021 Sep 3;21:213. doi: 10.1186/s12871-021-01427-1 (PMC8414757; doi:10.1186/s12871-021-01427-1)

Fig2K

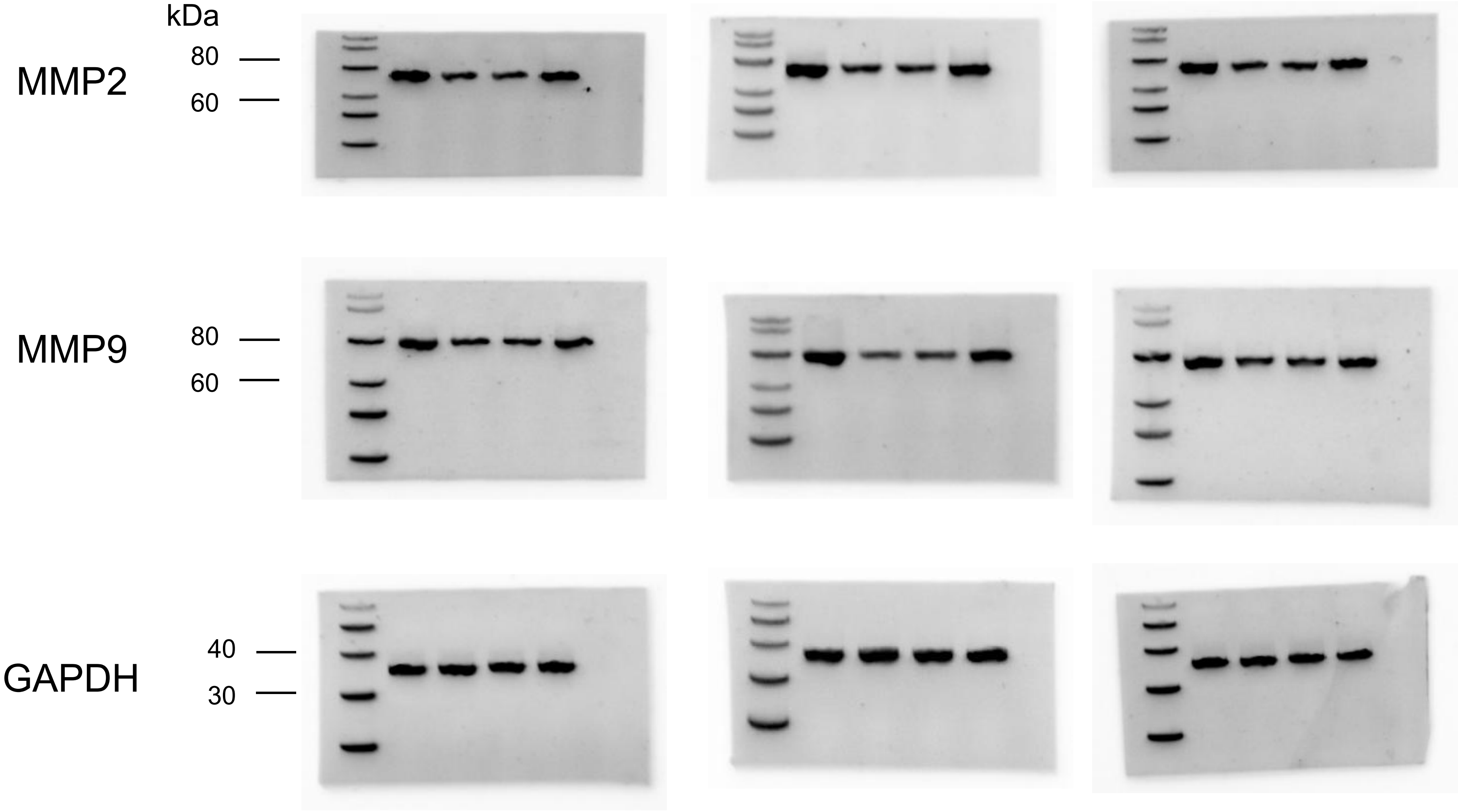

Fig2L

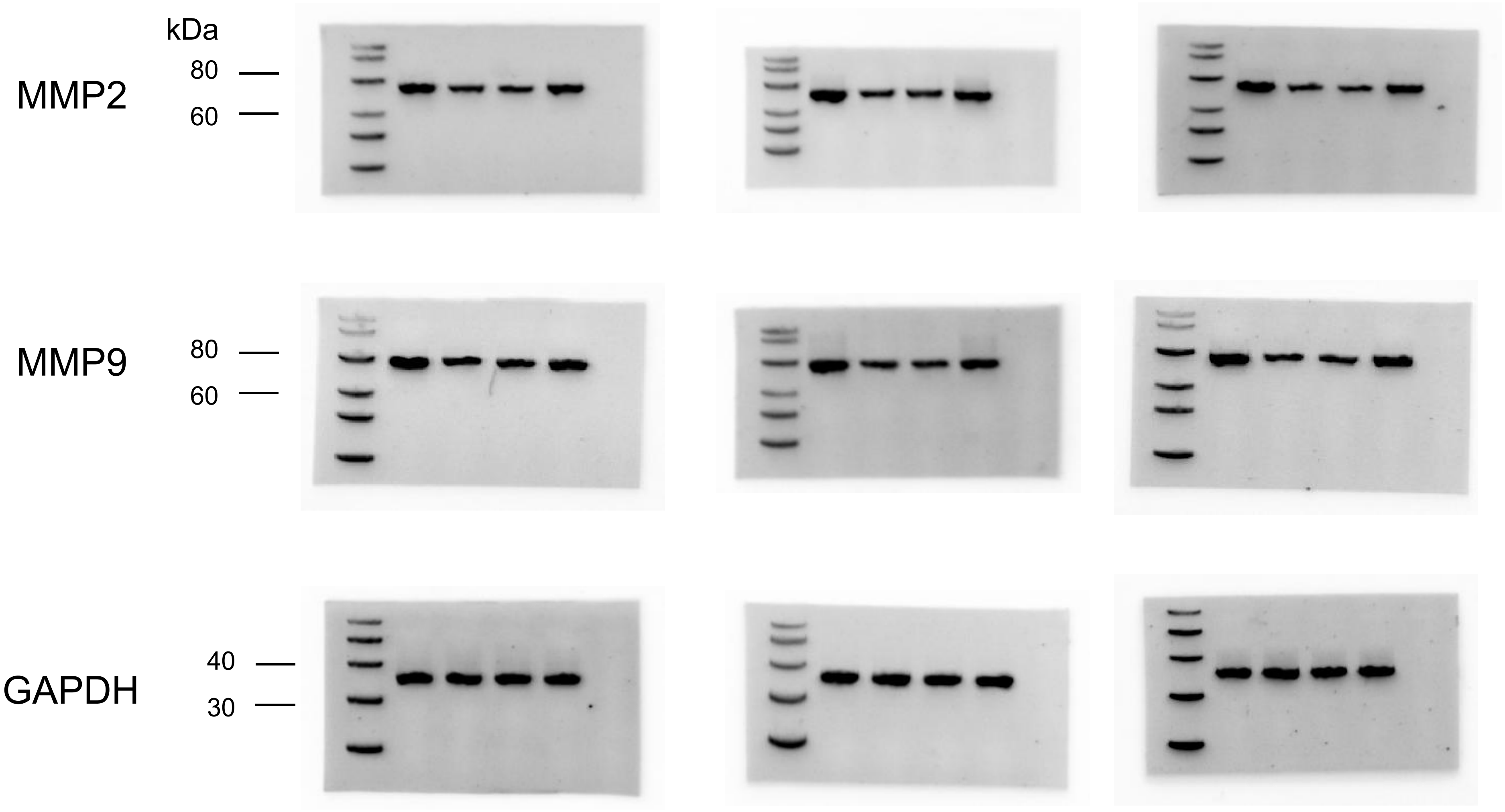

Fig4K

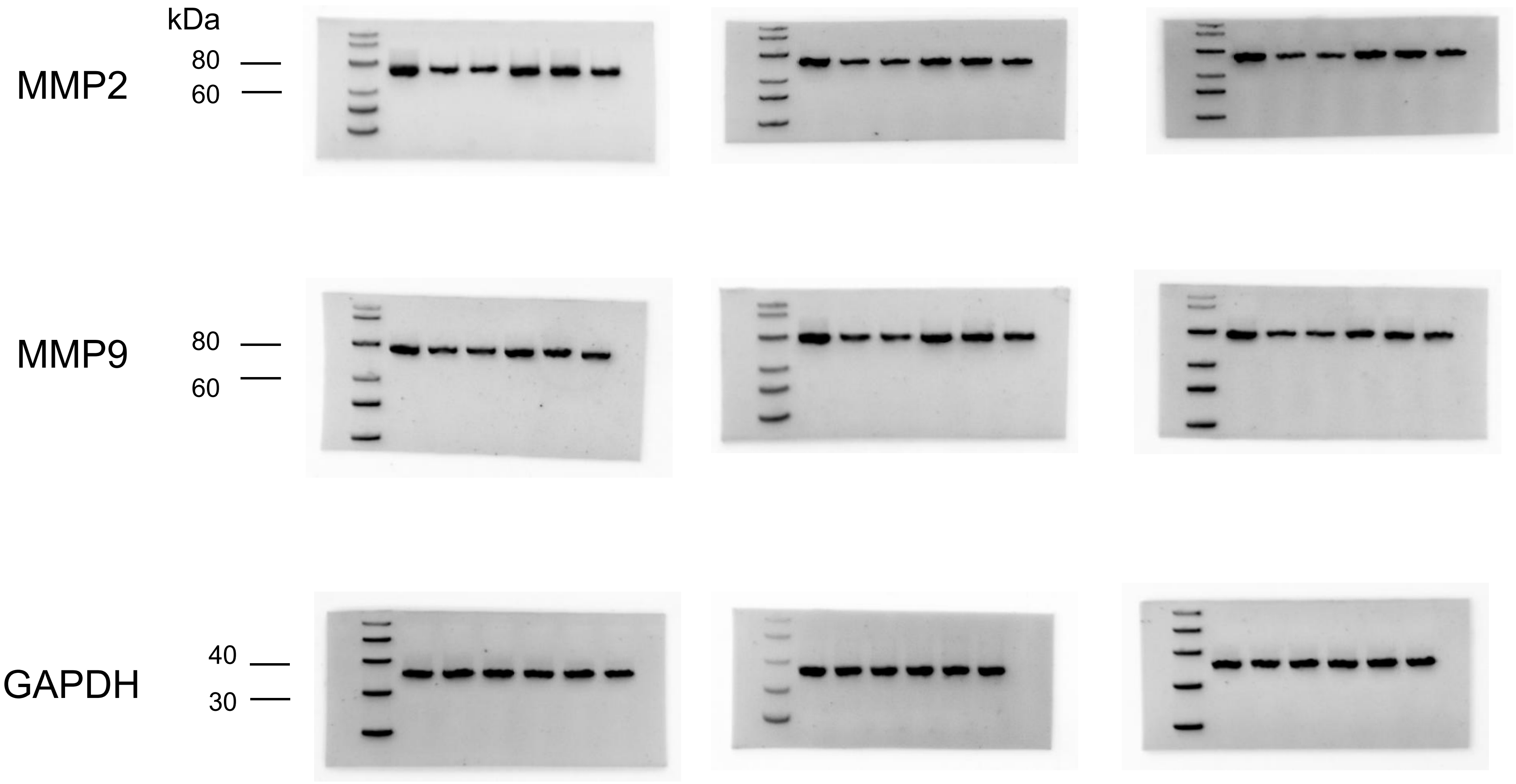

Fig4L

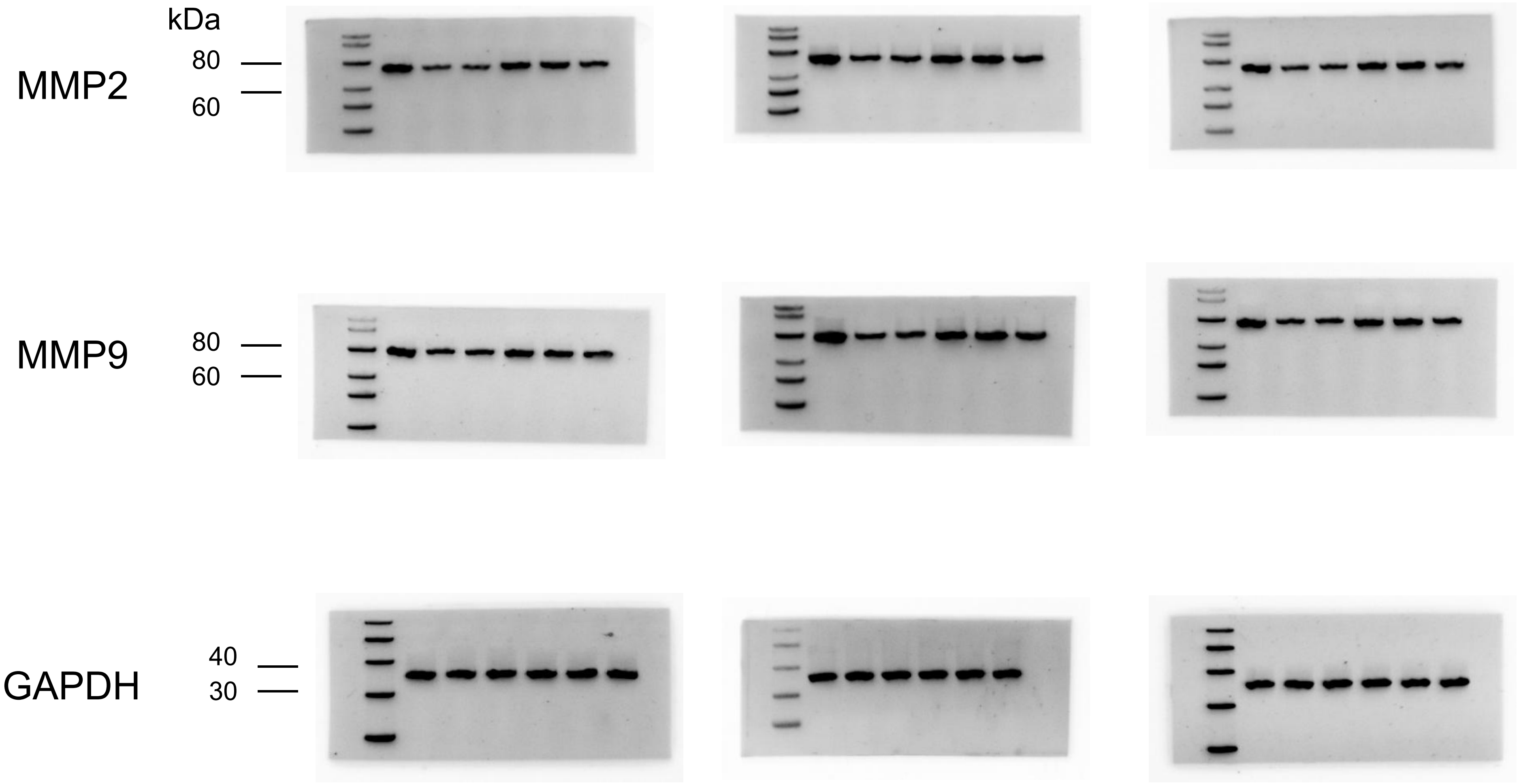

Fig5E

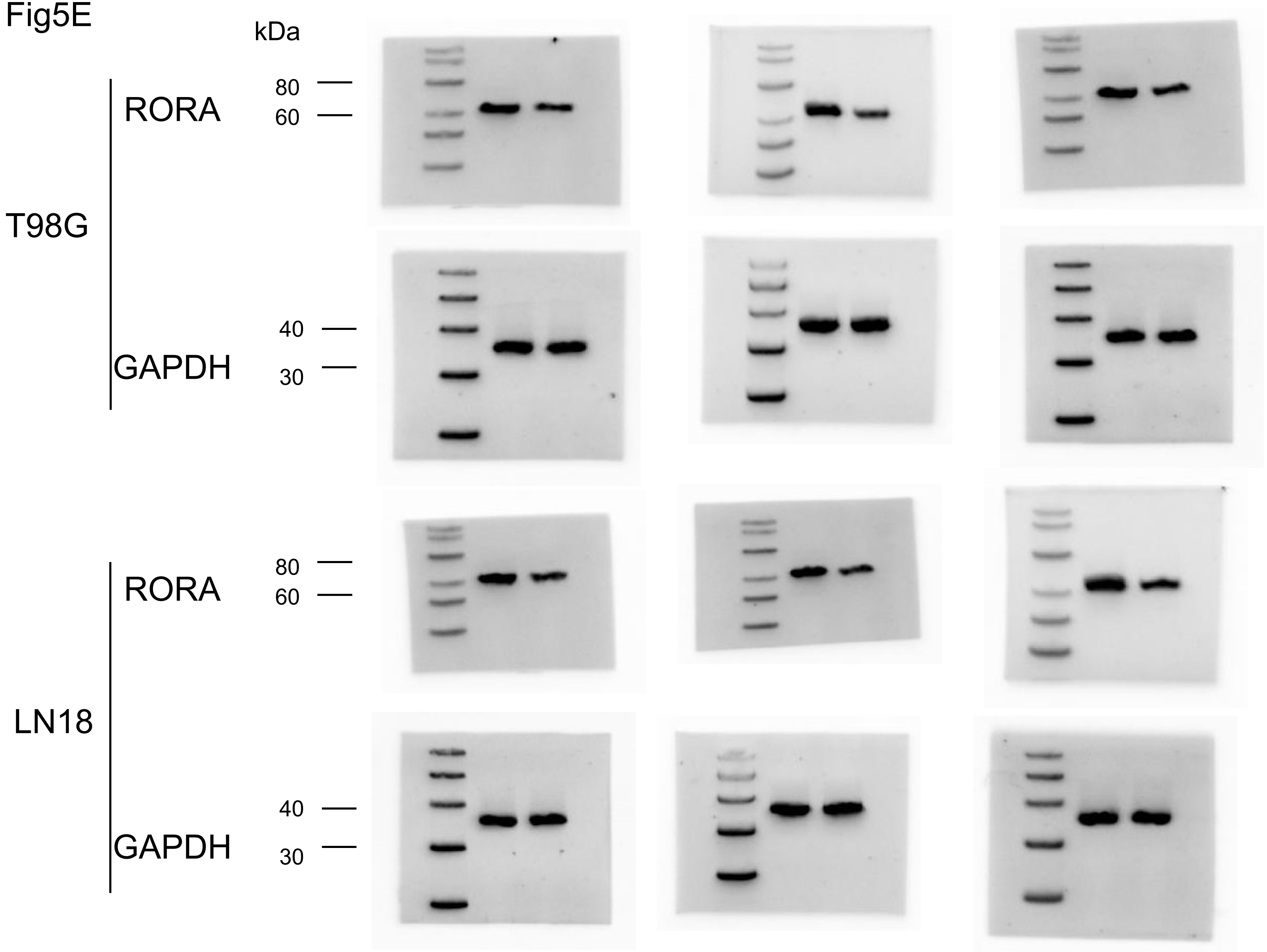

Fig5G

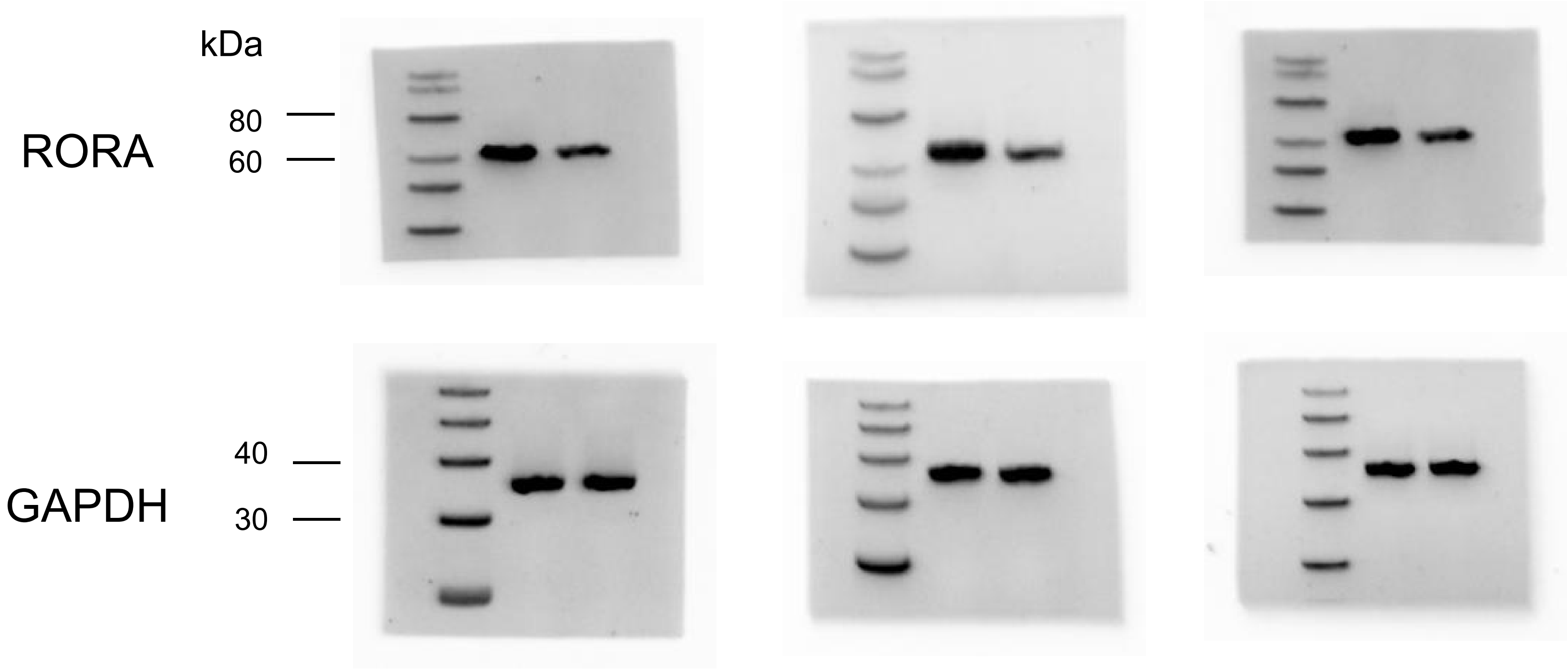

Fig5I

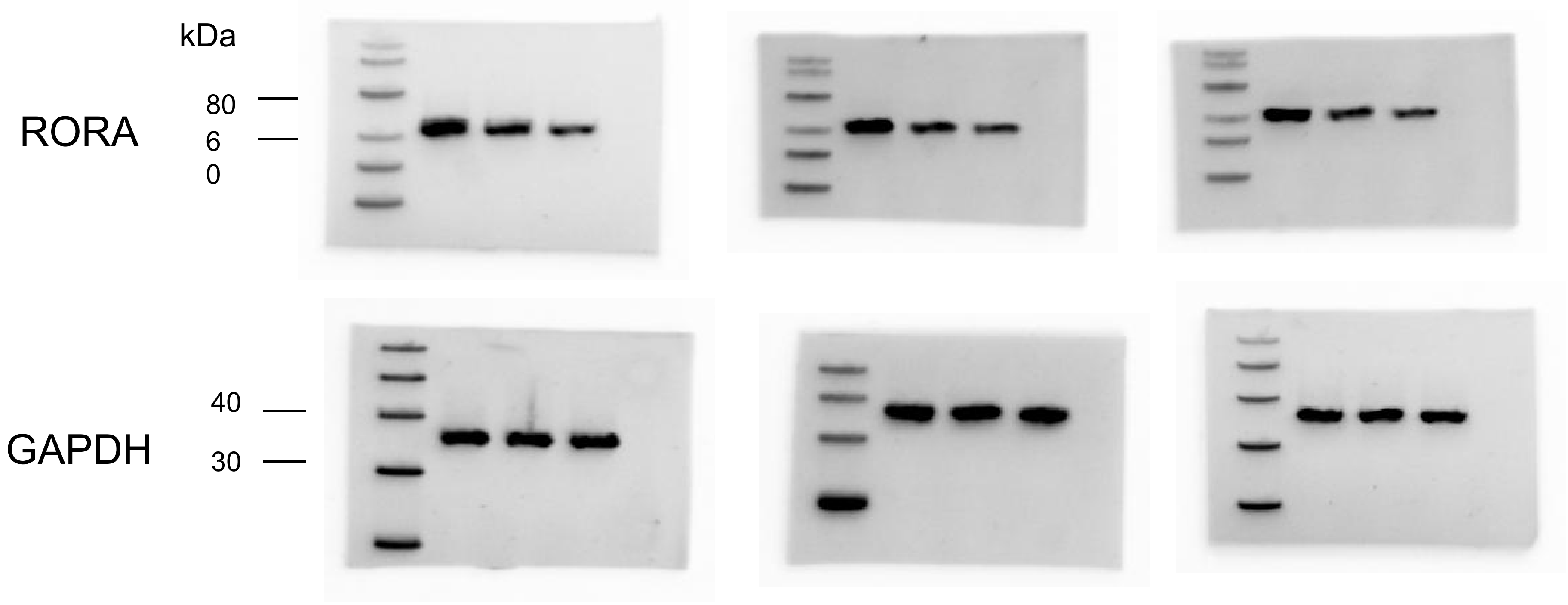

Fig5K

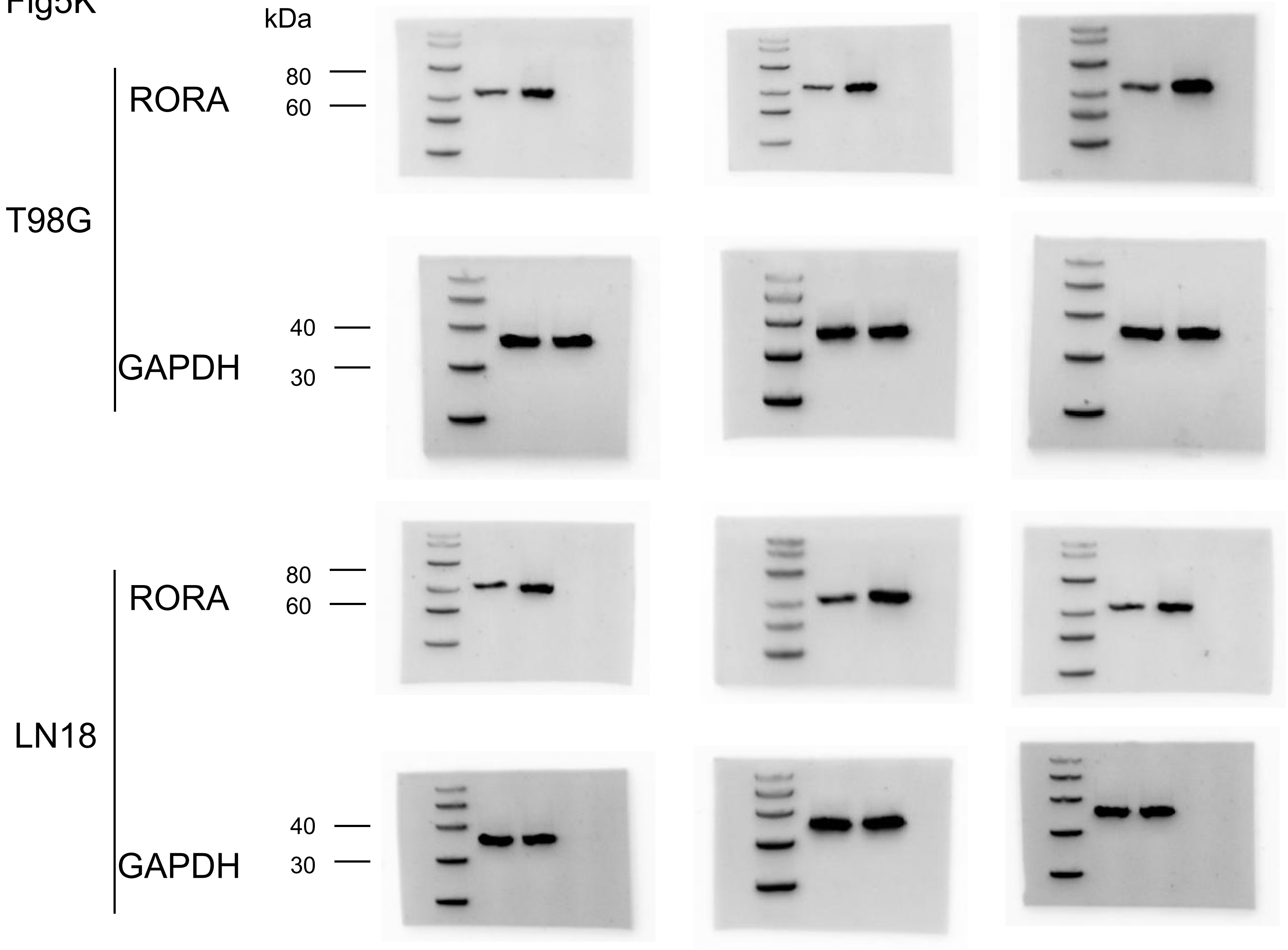

Fig5M

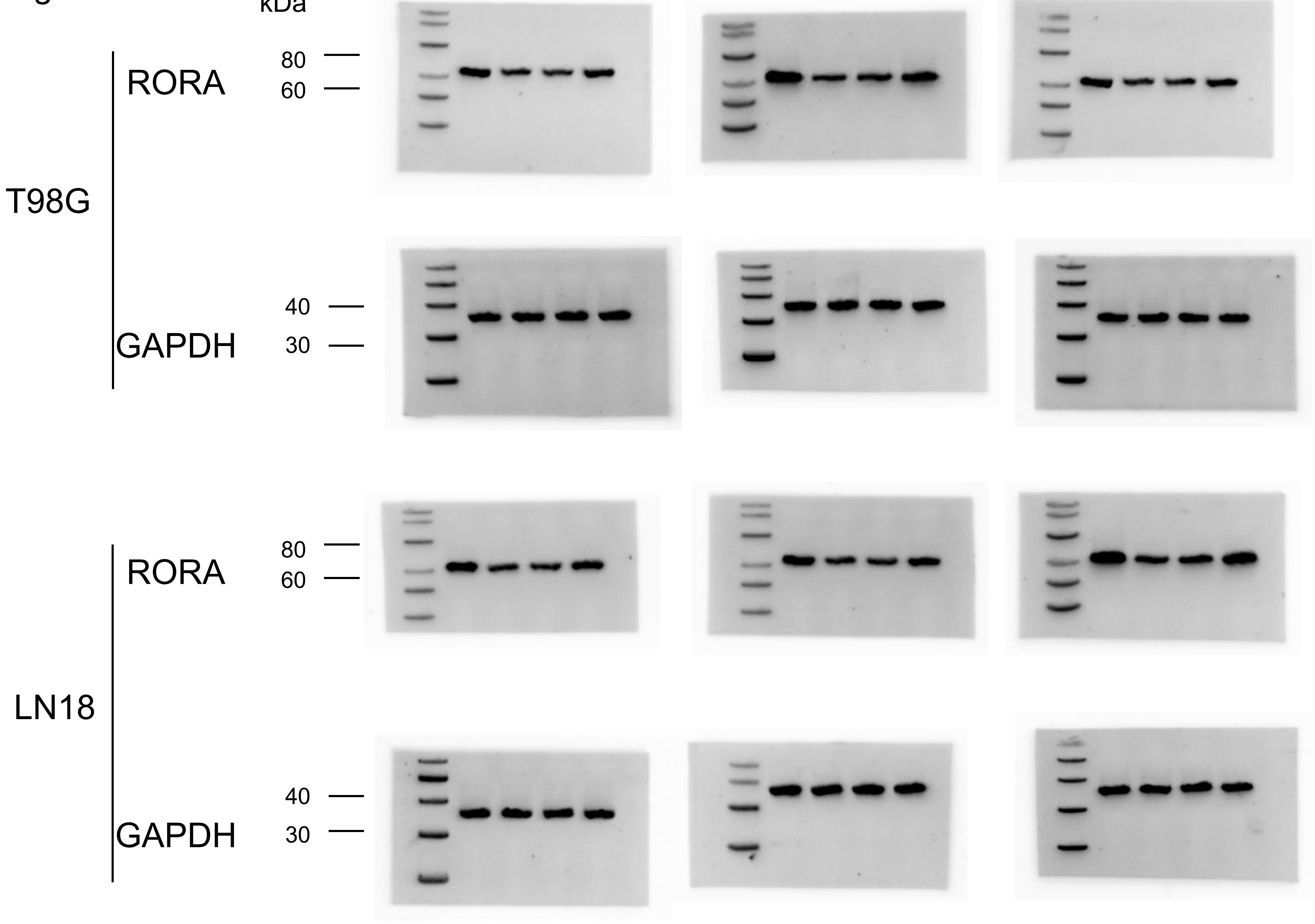

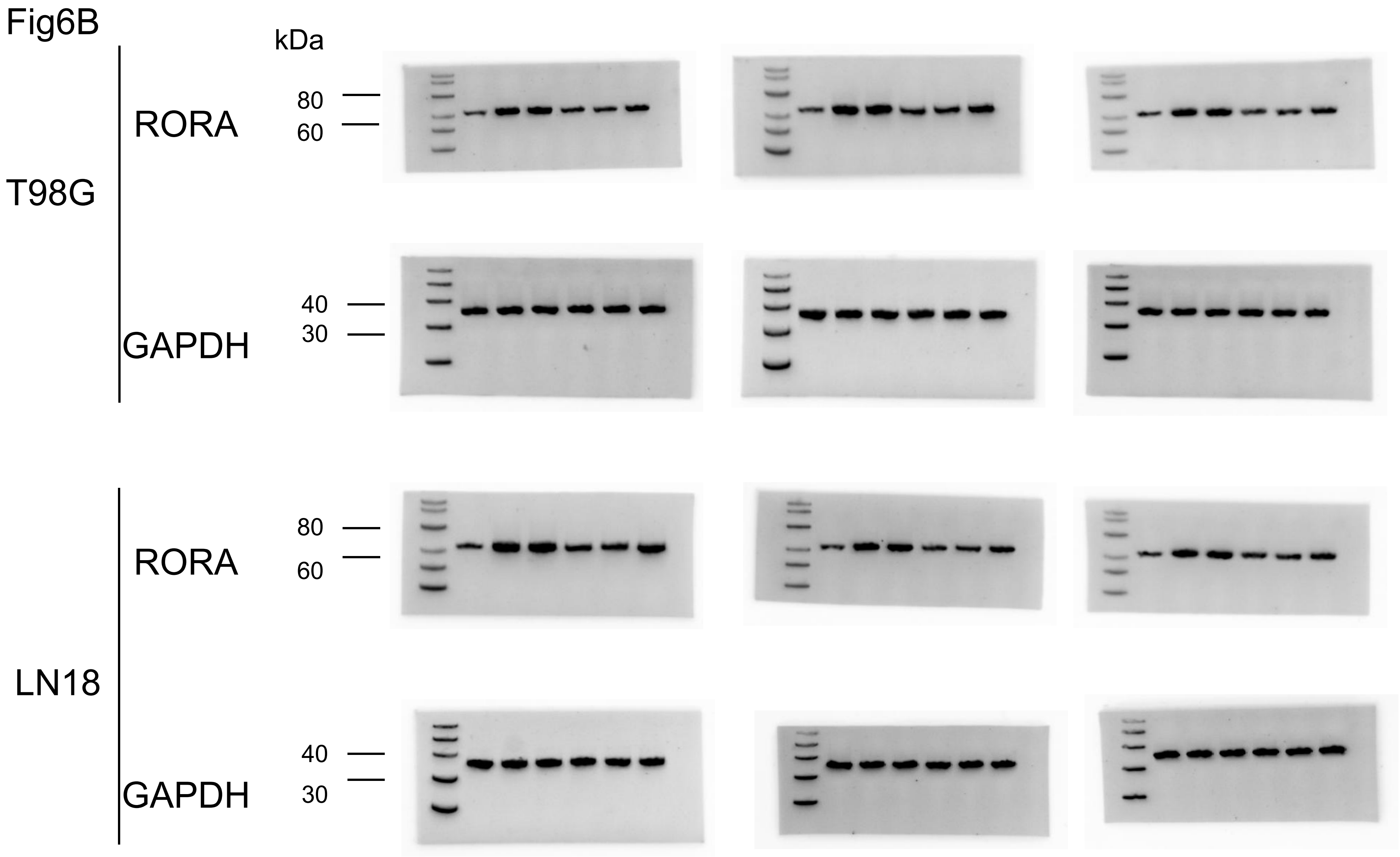
Fig6B
kDa
T98G
RORA
80
60
GAPDH
40
30
LN18
RORA
80
60
GAPDH
40
30

Fig6L

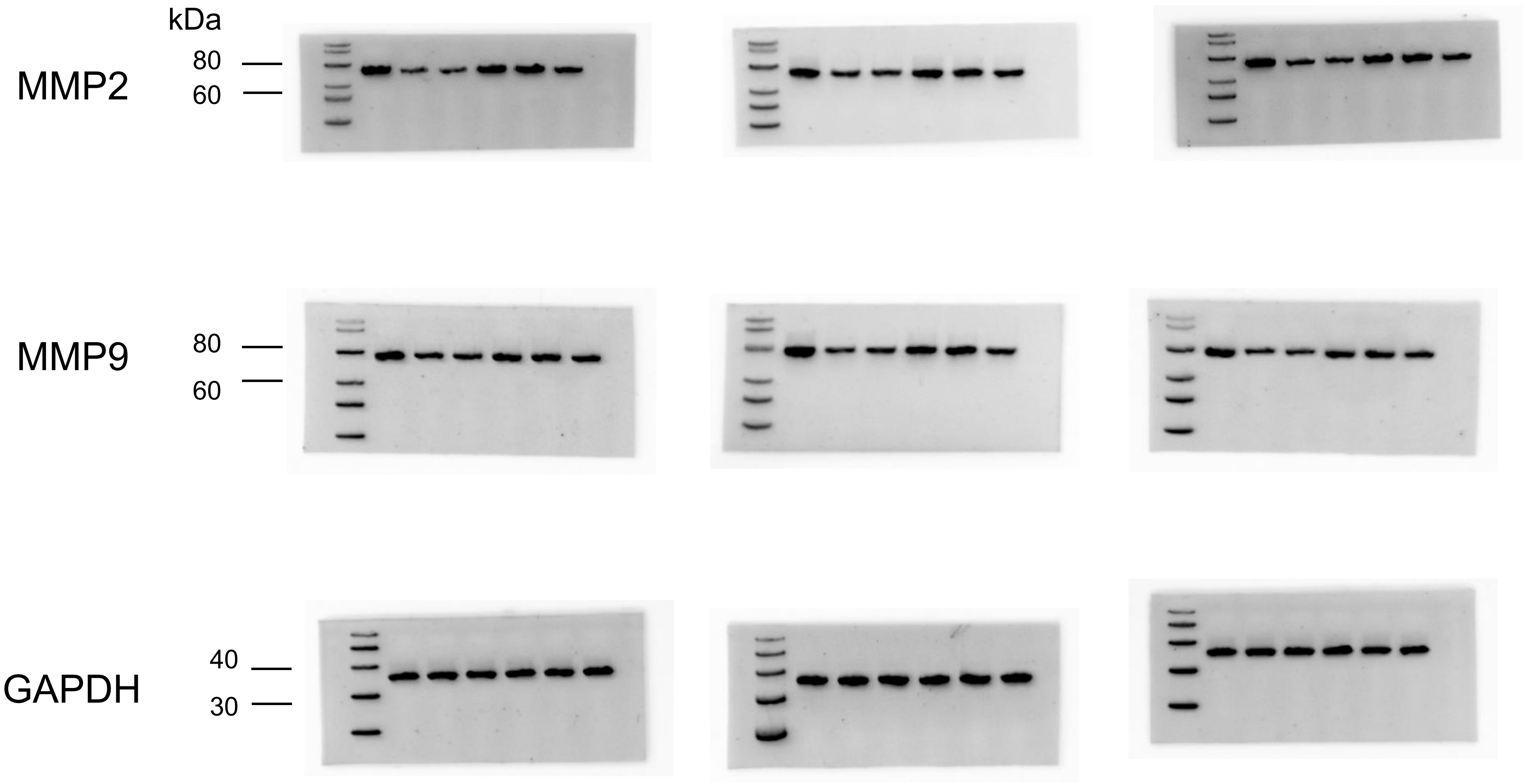

Fig6M

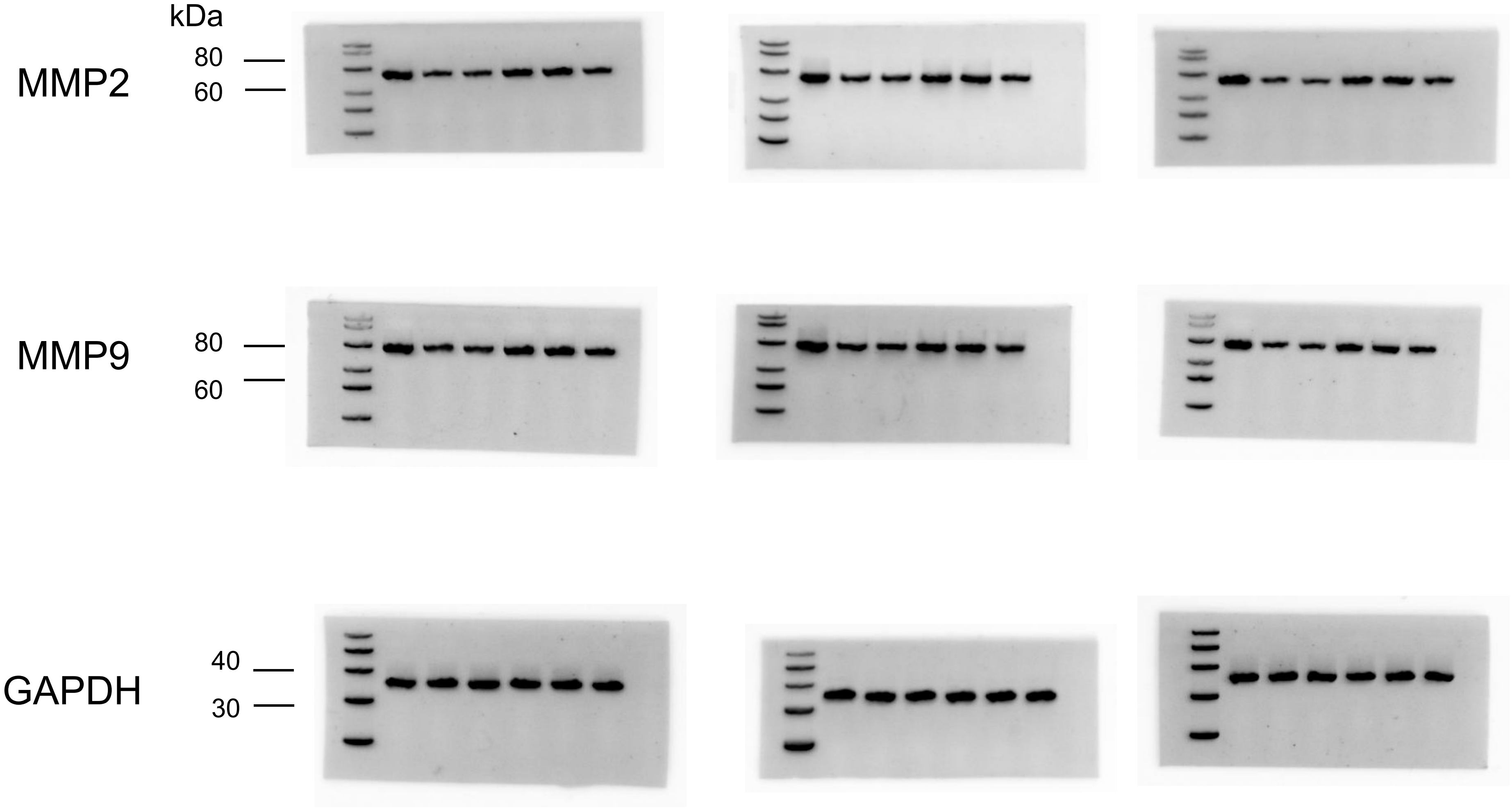

Fig7F

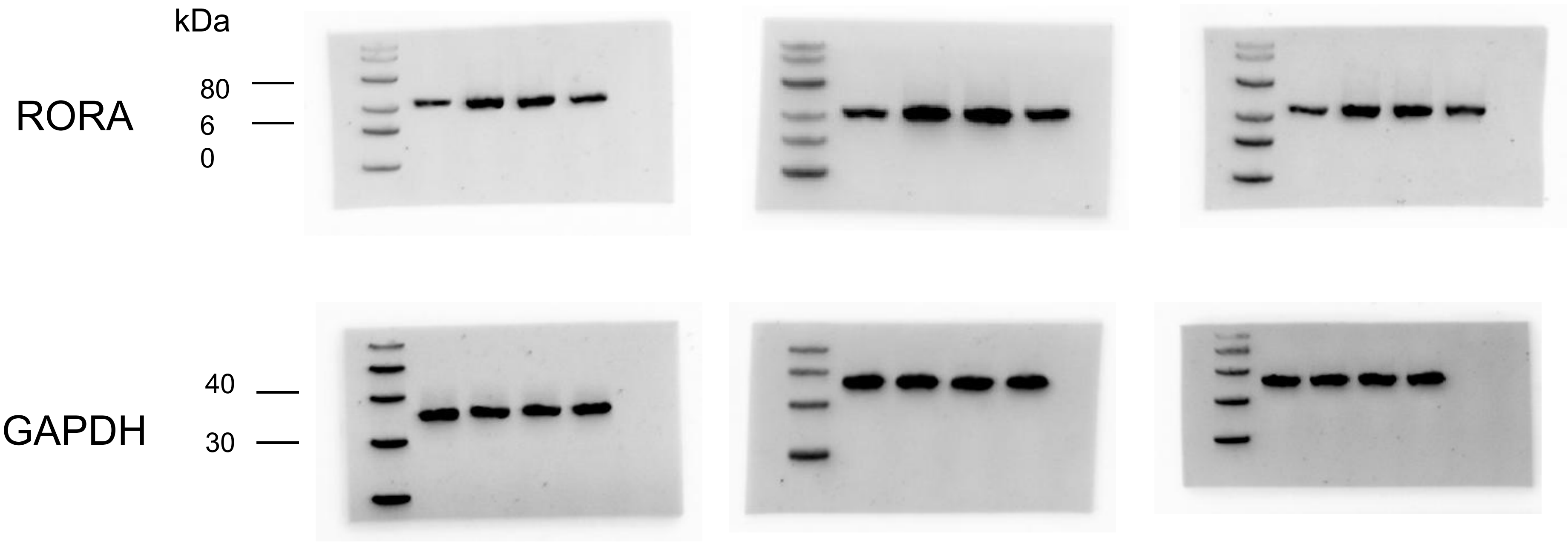

Supplement: Supplementary file 1 — Additional file 1. [file 12871_2021_1427_MOESM1_ESM.pdf]
